# Charmonium and charmoniumlike states at the BESIII experiment

**DOI:** 10.1093/nsr/nwab182

**Published:** 2021-10-05

**Authors:** Chang-Zheng Yuan

**Affiliations:** Institute of High Energy Physics, Chinese Academy of Sciences, Beijing 100049, China; University of Chinese Academy of Sciences, Beijing 100049, China

**Keywords:** charmonium states, charmoniumlike states, exotic hadrons, *e* ^+^ *e* ^−^ annihilation

## Abstract

Charmonium is a bound state of a charmed quark and a charmed antiquark, and a charmoniumlike state is a resonant structure that contains a charmed quark and antiquark pair but has properties that are incompatible with a conventional charmonium state. While operating at center-of-mass energies from 2 to 5 GeV, the BESIII experiment can access a wide mass range of charmonium and charmoniumlike states, and has contributed significantly in this field. We review BESIII results involving conventional charmonium states, including the first observation of the M1 transition ψ(2*S*) → γη_*c*_(2*S*) and the discovery of the ψ_2_(3823) state; and report on studies of charmoniumlike states, including the discoveries of the *Z*_*c*_(3900) and *Z*_*c*_(4020) tetraquark candidates, the resolution of the fine structure of the *Y*(4260) state, the discovery of the new production process *e*^+^*e*^−^ → γ*X*(3872) and the uncovering of strong evidence for the commonality among the *X*(3872), *Y*(4260) and *Z*_*c*_(3900) states. The prospects for further research at BESIII and proposed future facilities are also presented.

## Teaser

With the discovery of ‘four-quark matter’ Zc(3900) and Zc(4020), the observation of a new particle psi_2(3823), and the measurement of a very rare gamma-ray transition between charmonium states, the BESIII experiment opens fresh vista on matter.

## INTRODUCTION

In the conventional quark model, mesons are comprised of a quark and antiquark pair, while baryons are comprised of three quarks. A bound state of a charmed quark (*c*) and a charmed antiquark ($\bar{c}$) is named charmonium. The first charmonium state, the *J*/ψ, was discovered at BNL [1] and at SLAC [2] in 1974, and since then, all the charmonium states below the open-charm threshold and a few vector charmonium states above the open-charm threshold have been established [3]; the measured spectrum of states agrees well with theoretical calculations based on QCD [4–6] and QCD-inspired potential models [7–9].

In addition to the charmonium states, the current QCD-based theoretical framework describes almost all of the other hadrons that have been observed to date quite well, including three-quark baryons and other quark-antiquark mesons [3]. Exotic hadronic states with configurations not limited to two or three quarks have been the subject of numerous theoretical proposals and experimental searches [10,11]. These proposed exotic hadrons include hadron-hadron molecules, diquark-diantiquark tetraquark states, hadro-quarkonia, quark-antiquark-gluon hybrids, multi-gluon glueballs and pentaquark baryons.

Many charmonium and charmoniumlike states were discovered at the BaBar [12] and Belle [13] *B* factories during the first decade of this century [14]. While some of these are good candidates for conventional charmonium states, there are other states that have properties that do not match those of any of the unassigned $c\bar{c}$ states, which may indicate that exotic states have already been observed [5,15–17]. These candidate exotic meson states are collectively called the $\rm XYZ$ particles, to indicate that their underlying nature is still unclear. Although this is not fully accepted within the high energy physics community, practitioners in the field use *Z*_*Q*_(xxxx) to denote a quarkoniumlike state with mass roughly xxxx MeV/*c*^2^ that contains a heavy quark pair $Q\bar{Q}$ and with non-zero isospin, *Y*(xxxx) for a vector quarkoniumlike state (called ψ(xxxx) by PDG [3]) and *X*(xxxx) for states with other quantum numbers.

Although the BaBar [12] and Belle [13] experiments finished data taking in 2008 and 2010, respectively, the data are still used for various physics analyses. In 2008, two new experiments—BESIII [18], a τ-charm factory experiment at the BEPCII *e*^+^*e*^−^ collider, and LHCb [19], a *B*-factory experiment at the LHC *pp* collider—started data taking, and have been contributing to the study of charmonium and charmoniumlike states ever since.

The BESIII experiment at the BEPCII double ring *e*^+^*e*^−^ collider observed its first collisions in the τ-charm energy region in July 2008. The BESIII detector [18] is a magnetic spectrometer with an effective geometrical acceptance of 93% of 4π and state-of-the-art subdetectors for high precision charged and neutral particle measurements. After a few years of running at center-of-mass (c.m.) energies for its well-defined physics programs [20], i.e. at the *J*/ψ and ψ(2*S*) peaks in 2009 and the ψ(3770) peak in 2010 and 2011, the BESIII experiment began to collect data for the study of the $\rm XYZ$ particles, a program that was only mentioned tentatively in the BESIII Yellow Book [20]. The first data sample was collected at the ψ(4040) resonance in May 2011 with an integrated luminosity of about 0.5 fb^−1^. This sample was used to search for the production of the *X*(3872) and the excited *P*-wave charmonium spin-triplet states via ψ(4040) radiative transitions. The size of the sample was limited by the brief, one-month running time following the ψ(3770) data taking in the 2010–11 run.

In summer 2012, the LINAC of the BEPCII was upgraded so that the highest beam energy was increased from 2.1 to $2.3\,\rm GeV$, which made it possible to collect data at higher c.m. energies (up to $4.6\,\,\rm GeV$). A data sample of 525 pb^−1^ was collected at a c.m. energy of $4.26\,\,\rm GeV$ from 14 December 2012 to 14 January 2013, with which the *Z*_*c*_(3900) charged charmoniumlike state was discovered [21]. This observation changed the data collection plan for the 2012–13 run and had considerable impact on the subsequent running schedule of the experiment; more data points between 4.13 and $4.60\,\,\rm GeV$ dedicated to the $\rm XYZ$ related analyses were recorded [22]. The highest beam energy was further increased from 2.3 to $2.5\,\,\rm GeV$ in summer 2019, making it possible to collect data at even higher c.m. energies (up to $5.0\,\,\rm GeV$).

The data samples used for the $\rm XYZ$ study cover the energy range between 4.0 and 5.0 GeV, with a typical integrated luminosity of 500 pb^−1^ at each energy point. These data were also used for charmonium studies together with a 448 million ψ(2*S*) event sample. Data samples with an integrated luminosity of 826 pb^−1^ at 104 energy points between 3.8 and $4.6\,\,\rm GeV$ [23] were also used for the $\rm XYZ$ study.

In this article, we review studies of charmonium and charmoniumlike states from the BESIII [18] experiment. We first introduce the study of conventional charmonium states and then the $\rm XYZ$ states. Finally, we discuss prospects for future studies with the BESIII experiment, and also point out possible studies at next generation facilities.

## CONVENTIONAL CHARMONIUM STATES

The search for new charmonium states has always been a high priority topic. With the data taken at c.m. energies above 4 GeV, it is possible to search for states predicted by theories that are still unobserved [4–9]. These states include the excited *P*-wave spin-triplet states χ_*cJ*_(2*P*) (*J* = 0, 1, 2), the excited *P*-wave spin-singlet state *h*_*c*_(2*P*), the *D*-wave spin-triplet states ψ_*J*_(1*D*) (*J* = 2, 3; the *J* = 1 state, the ψ(3770), was observed many years ago [3]) and the *D*-wave spin-singlet state η_*c*2_(1*D*).

The predicted mass of the *D*-wave charmonium states (excluding the ψ(3770), which is, in fact, a mixture of the 1 ^3^*D*_1_ and 2 ^3^*S*_1_ vector states) is in the 3.81–3.85 GeV/*c*^2^ range predicted by several phenomenological calculations [7–9]. Since the mass of ψ_2_(1*D*) is above the $D\bar{D}$ threshold but below the $D\bar{D}^*$ threshold, and $\psi _2(1D)\rightarrow D\bar{D}$ violates parity, the ψ_2_(1*D*) is expected to be narrow and its dominant decay mode is ψ_2_(1*D*) → γχ_*c*1_ [24]. The ψ_2_(1*D*) state, also called the ψ_2_(3823), was discovered at BESIII [25] in this final state, and the ψ_3_(1*D*) state was observed by LHCb in its decay into the $D\bar{D}$ final state [26].

The spin-triplet charmonium states are produced copiously in *e*^+^*e*^−^ annihilation and in *B* decays and, thus, they are understood much better than the spin-singlet charmonium states, including the lowest lying *S*-wave state, the η_*c*_, its radial excited partner, the η_*c*_(2*S*), and the *P*-wave spin-singlet state, the *h*_*c*_. Since these three states are all produced in ψ(2*S*) decays, the world’s largest ψ(2*S*) data sample at BESIII made it possible to study their properties with improved precision. In addition, the unexpected large production cross section for *e*^+^*e*^−^ → π^+^π^−^*h*_*c*_ in the BESIII energy region [27] opened a new mechanism for studying the *h*_*c*_ and η_*c*_ (from *h*_*c*_ → γη_*c*_), and BESIII contributed the world’s best measurements of the properties of these states [28]. We report here the observation of the M1 transition ψ(2*S*) → γη_*c*_(2*S*) at BESIII [29], a transition that has been sought for since the first-generation BES experiment in the 1980s.

### Discovery of the M1 transition **ψ**(2*S*) → **γη**_*c*_(2*S*)

The production of η_*c*_(2*S*) through a radiative transition from ψ(2*S*) involves a charmed-quark spin-flip and, thus, proceeds via a magnetic dipole (M1) transition. The branching fraction has been calculated by many authors, with predictions in the range ${\cal B}(\psi (2S)\rightarrow \gamma \eta _c(2S))= (0.1\!-\!6.2)\times 10^{-4}$ [30–32]. Experimentally, this transition has been searched for by Crystal Ball [33], BES [34,35] and CLEO [36]. No convincing signal was observed in any of these experiments.

**Figure 1.**
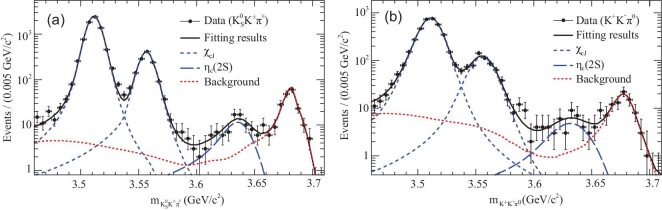
The fit to the invariant-mass spectra for (a) $K_{S}^{0}K^\pm \pi ^\mp$ and (b) *K*^+^*K*^−^π^0^ [29]. Dots with error bars are data, and the curves are total fits and individual components. The lowest peaks correspond to the η_*c*_(2*S*) signals.

With a sample of 106 million ψ(2*S*) events collected at BESIII, the process ψ(2*S*) → γη_*c*_(2*S*) was observed for the first time with $\eta _c(2S)\rightarrow K_{S}^{0}K^\pm \pi ^\mp$ and *K*^+^*K*^−^π^0^ decay modes. The final $K\bar{K}\pi$ mass spectra and the fit results are shown in Fig. 1. For the number of η_*c*_(2*S*) signal events, the fit yields 81 ± 14 for the $K_{S}^{0}K^\pm \pi ^\mp$ mode and 46 ± 11 for the *K*^+^*K*^−^π^0^ mode; the overall statistical significance of the signal is larger than 10σ [29].

The mass of η_*c*_(2*S*) is measured to be (3637.6 ± 2.9 ± 1.6) MeV/*c*^2^, the width (16.9 ± 6.4 ± 4.8) MeV, in good agreement with the PDG world average values [3], and the product branching fractions ${\cal B}(\psi (2S)\rightarrow \gamma \eta _c(2S))\times {\cal B}(\eta _c(2S)\rightarrow K\bar{K}\pi ) = (1.30\pm 0.20\pm 0.30)\times 10^{-5}$. Combining the production rate with a BaBar measurement of ${\cal B}(\eta _c(2S)\rightarrow K\bar{K} \pi )$, the M1 transition rate is determined to be ${\cal B}(\psi (2S)\rightarrow \gamma \eta _c(2S)) = (6.8\pm 1.1_\mathrm{stat}\pm 4.5_\mathrm{sys})\times 10^{-4}$. This agrees with theoretical calculations [30–32] and naive estimates based on the *J*/ψ → γη_*c*_ transition [36].

This study benefited from the BESIII detector’s high resolution electromagnetic calorimeter, which makes the detection of the radiative photon with 50 MeV energy possible [18]. Given the tiny transition rate and the low photon energy, it is understandable why this transition was not observed in previous studies [33,34,36]. This is the third M1 transition observed in a charmonium system (the other two are *J*/ψ → γη_*c*_ and ψ(2*S*) → γη_*c*_ observed in 1980 [37]); improved measurements of these transitions and discovery of more M1 transitions would improve the understanding of the high-order effects involved in these transitions [9,38,39].

### Observation of the **ψ**_2_(1*D*) state

**Figure 2.**
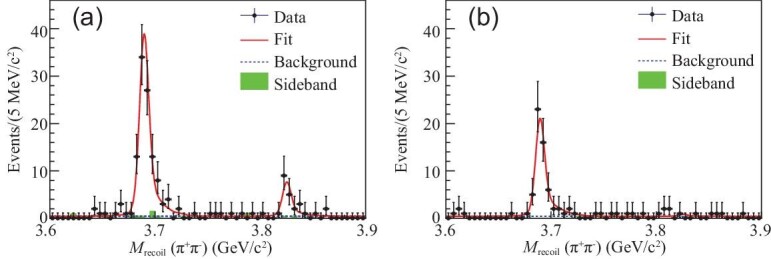
Simultaneous fit to the *M*_recoil_(π^+^π^−^) distribution of (a) γχ_*c*1_ events and (b) γχ_*c*2_ events [25]. The small peak in (a) is the ψ_2_(3823) signal. Dots with error bars are data, red solid curves are total fits, dashed blue curves are background fits and the green shaded histograms are *J*/ψ mass sideband events.

The processes of *e*^+^*e*^−^ → π^+^π^−^γχ_*c*1, 2_ are studied at the BESIII experiment using 4.1 fb^−1^ of data collected at c.m. energies from 4.23 to $4.60\,\,\rm GeV$ [25]. The χ_*c*1, 2_ are reconstructed via their decays into γ*J*/ψ, with *J*/ψ to ℓ^+^ℓ^−^ (ℓ = *e*, μ). A clear signal is observed as a 19 ± 5 event peak in the γχ_*c*1_ invariant mass distribution that is evident in Fig. 2(a). The statistical significance of the signal is 6.2σ, its mass is determined to be (3821.7 ± 1.3 ± 0.7) MeV/*c*^2^ and its properties are in good agreement with the ψ_2_(1*D*) charmonium state. The state is thus called the ψ_2_(3823) following the 3.8σ ‘evidence’ in *B* decays reported by Belle [40] in 2013. For the γχ_*c*2_ mode, no significant ψ_2_(3823) signal is observed (Fig. 2(b)), and an upper limit on its production rate is determined. BESIII obtains the ratio ${{\cal B}[\psi _2(3823)\rightarrow \gamma \chi _{c2}]} /{{\cal B}[\psi _2(3823)\rightarrow \gamma \chi _{c1}]}<0.42$ at the 90% confidence level (C.L.), which also agrees with expectations for the ψ_2_(1*D*) state [24].

With the observation of three *D*-wave spin-triplet states (ψ(3770), ψ_2_(3823) and ψ_3_(3842)), their center of gravity, 3822 MeV/*c*^2^, is a good estimation of the mass of the *D*-wave spin-singlet state, η_*c*2_(1*D*). Since it cannot decay into open-charm final states, η_*c*2_(1*D*) is expected to be very narrow, and the identification of it should be clear, if it is produced with large enough rate, in *e*^+^*e*^−^ → γη_*c*2_(1*D*), *e*^+^*e*^−^ → ωη_*c*2_(1*D*) or *e*^+^*e*^−^ → π^+^π^−^*h*_*c*_(2*P*) → π^+^π^−^γη_*c*2_(1*D*).

## EXOTIC CHARMONIUMLIKE STATES

A revival of the study of charmonium spectroscopy occurred in the early twenty-first century when the BaBar and Belle *B* factories started accumulating large data samples at the ϒ(4*S*) peak. The high luminosity at these *B* factories enabled studies of charmonium states that are produced in a variety of ways, including *B* decays, initial-state-radiation (ISR) processes, double-charmonium production, two-photon processes, etc. While the discovery of the conventional charmonium states such as η_*c*_(2*S*) and χ_*c*2_(2*P*) were more-or-less routine, the observations of the *X*(3872) by Belle in 2003 [41] and the *Y*(4260) by BaBar in 2005 [42], the first of the $\rm XYZ$ mesons, came as big surprises; although these new states decay to final states that contain both a *c* and a $\bar{c}$ quark, they have properties that do not match those of any $c\bar{c}$ meson [5,15–17].

All studies of $\rm XYZ$ states at the *B* factories have low statistics and limited precision. In contrast, BESIII can tune the c.m. energy to match the peaks of the *Y* states, where event rates are high enough to facilitate precise measurements of their resonance parameters and search for new states among their decay products.

### New insights into the *Y* states

The *Y* states, such as *Y*(4260) [42], *Y*(4360) [43,44] and *Y*(4660) [44], are produced directly or via the ISR process in *e*^+^*e*^−^ annihilation and, thus, are vectors with quantum numbers *J*^*PC*^ = 1^−−^. These states have strong couplings to hidden-charm final states in contrast to the established vector charmonium states in the same mass region, such as ψ(4040), ψ(4160) and ψ(4415), which dominantly couple to open-charm meson pairs [45,46].

In potential models, five vector charmonium states are expected to be in the mass region between 4.0 and 4.7 GeV/*c*^2^, namely ψ(3*S*), ψ(2*D*), ψ(4*S*), ψ(3*D*) and ψ(5*S*), with the first three identified with the well-established ψ(4040), ψ(4160) and ψ(4415) charmonium mesons; the masses of the as yet undiscovered ψ(3*D*) and ψ(5*S*) are expected to be higher than 4.4 GeV/*c*^2^. However, six vector states in this mass region have been identified, as listed above. These make the *Y*(4260), *Y*(4360) and perhaps the *Y*(4660) states good candidates for new types of exotic particles, stimulating many theoretical interpretations, including tetraquark states, molecular states, hybrid states or hadro-charmonia [5,15–17].

The *Y*(4260) was first observed at the *B* factories as a distinct peak in the π^+^π^−^*J*/ψ invariant mass distribution for ISR-produced *e*^+^*e*^−^ → γ_ISR_π^+^π^−^*J*/ψ events [42,47]. Improved measurements from both BaBar [48] and Belle [49] with their full data samples confirmed the existence of both the *Y*(4260) resonance and a non-*Y*(4260)-resonance component in *e*^+^*e*^−^ → π^+^π^−^*J*/ψ around $4.0\,\,\rm GeV$, but the line shape was parameterized with different models. The parameters of the *Y*(4260) determined by fit to the combined data from the two *B*-factory experiments and the CLEO measurements [50] are *M*_*Y*(4260)_ = (4251 ± 9) MeV/*c*^2^ and $\Gamma _{Y(4260)}=(120\pm 12)\,\,\rm MeV$ [51]. High precision BESIII measurements of the direct cross section for the *Y*(4260) production in different final states supply new insight into its nature. These measurements include: *e*^+^*e*^−^ → π^+^π^−^*J*/ψ [52], *e*^+^*e*^−^ → π^+^π^−^*h*_*c*_ [27], *e*^+^*e*^−^ → ωχ_*cJ*_ [53,54], *e*^+^*e*^−^ → *D*^0^D^* −^π^+^ + c.c. [55], etc. [56].

**Figure 3.**
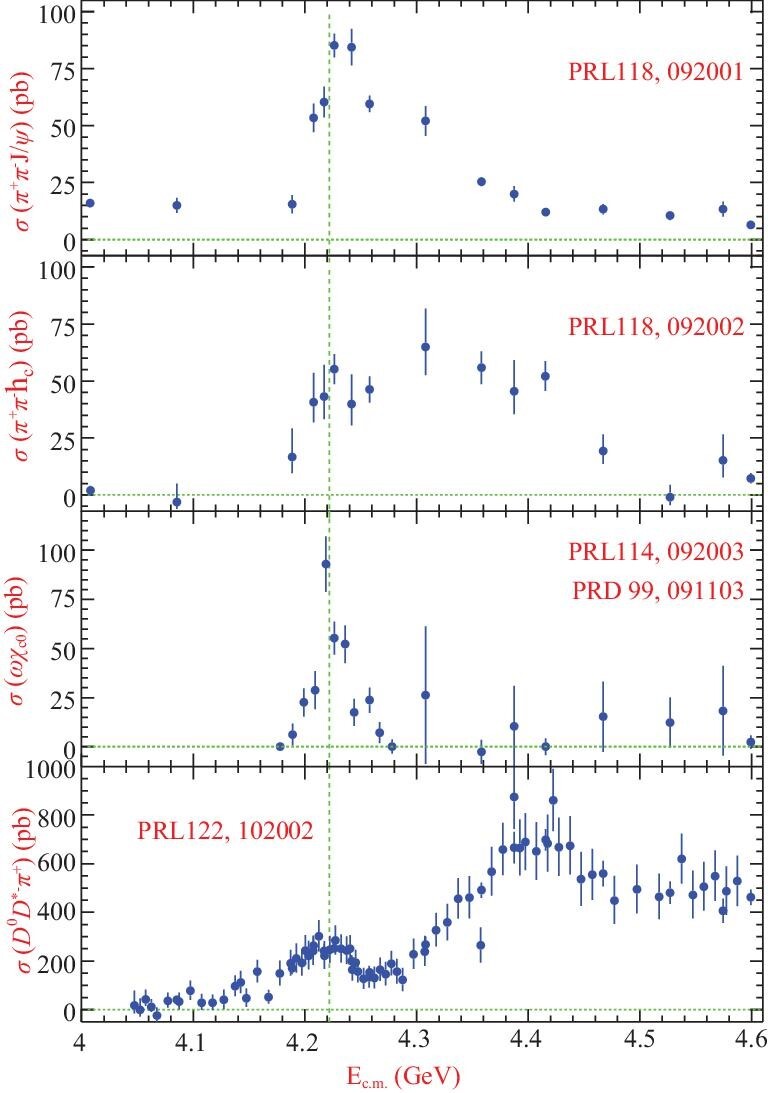
From top to bottom are the measured cross sections of *e*^+^*e*^−^ → π^+^π^−^*J*/ψ [52], *e*^+^*e*^−^ → π^+^π^−^*h*_*c*_ [27], *e*^+^*e*^−^ → ωχ_*c*0_ [53,54] and *e*^+^*e*^−^ → *D*^0^D^* −^π^+^ + c.c. [55] as a function of the center-of-mass energy (E_c.m._). Dots with error bars are the data and the dotted vertical line is the peak of the *Y*(4220).

Figure 3 shows the measured cross sections for each of these final states. The *Y*(4260) structure is evident, but its line shape is in fact not well described by a single Breit-Wigner (BW) resonance function. Instead, its line shape is peaked at around $4.22\,\,\rm GeV$, which is substantially lower than the average value from previous measurements [51], and a distinct shoulder is observed on its high-mass side that is especially pronounced in the π^+^π^−^*J*/ψ mode. In order to describe this line shape, two resonant structures in the *Y*(4260) peak region are needed. The lower one has a mass of (4222.0 ± 3.1 ± 1.4) MeV/*c*^2^ and a width of $(44.1\pm 4.3\pm 2.0)\,\,\rm MeV$, while the higher one has a mass of (4320.0 ± 10.4 ± 7.0) MeV/*c*^2^ and a width of $(101.4^{+25.3}_{-19.7}\pm 10.2)\,\,\rm MeV$. The mass of the first resonance is ∼30 MeV/*c*^2^ lower than the world average value at that time [51] for *Y*(4260) and its width is about a factor of 3 narrower. The second resonance is observed in the *e*^+^*e*^−^ → π^+^π^−^*J*/ψ process for the first time. It is still not clear whether it is a new state or just a new decay mode of the *Y*(4360) observed in *e*^+^*e*^−^ → π^+^π^−^ψ(2*S*) [43,44]. The resonance parameters for the lower mass structure, the *Y*(4220), are also measured in other decay channels and listed in Table 1.

**Table 1.** Resonance parameters of the *Y*(4220) state from different modes measured at BESIII. The cross sections measured at a c.m. energy of 4.226 GeV are also listed.

	Mass	Width	σ at $\sqrt{s}=4.226$ GeV
Mode	(MeV/*c*^2^)	(MeV)	(pb)
*e* ^+^ *e* ^−^ → π^+^π^−^*J*/ψ	4222.0 ± 3.1 ± 1.4	44.1 ± 4.3 ± 2.0	85.1 ± 1.5 ± 4.9
*e* ^+^ *e* ^−^ → π^+^π^−^*h*_*c*_	$4218.4^{+5.5}_{-4.5}\pm 0.9$	$66.0^{+12.3}_{-8.3}\pm 0.4$	55.2 ± 2.6 ± 8.9
*e* ^+^ *e* ^−^ → ωχ_*c*0_	4218.5 ± 1.6 ± 4.0	28.2 ± 3.9 ± 1.6	55.4 ± 6.0 ± 5.9
*e* ^+^ *e* ^−^ → π^+^*D*^0^D^* −^ + c.c.	4228.6 ± 4.1 ± 6.3	77.0 ± 6.8 ± 6.3	252 ± 5 ± 15

Since the resonant structure around 4.2 GeV/*c*^2^ is present in all of the above channels with similar resonance parameters, Gao *et al.* [57] applied a combined fit to the measured cross sections to determine the resonance parameters of the low-mass *Y*(4220) peak with a resultant mass of (4219.6 ± 3.3 ± 5.1) MeV/*c*^2^ and width of $(56.0 \pm 3.6 \pm 6.9)\,\,\rm MeV$. These values are very different from those obtained in previous experiments [51]. The fit also gives the product of the leptonic decay width and the decay branching fraction to the considered final state. After accounting for the unmeasured isospin partners of the measured channels, a lower limit on the leptonic partial width of the *Y*(4220) is determined to be $\Gamma _{e^+e^-} > (29.1\pm 7.4)\,\,\rm eV$, where the error is the combined fit error and those from different fit scenarios. Cao *et al.* [58] analyzed BESIII, Belle and BaBar data on charmonium as well as open-charm final states, and a leptonic width of *O*(0.1–1) keV was obtained. This partial width is much larger than LQCD predictions for a hybrid vector charmonium state [59].

In spite of the limited experimental information that has been available between the time of its discovery in 2005 and the recent BESIII measurements, the *Y*(4260) has attracted considerable attention. The BESIII measurements of its production, decay and line shape in a variety of final states enable more sophisticated theoretical investigations, and some analyses have been performed, such as those in [58] and those quoted in [5,15–17]. The presence of the nearby $D_s^{*+}D_s^{*-}$, $D\bar{D}_1(2420)$ and ωχ_*cJ*_ production thresholds, and its mass overlap with the ψ(4160) and ψ(4415) conventional charmonium states complicate its interpretation. Joint experimental and theoretical efforts will likely be required to gain a full understanding of the nature of this state; these include precision measurements of the cross sections of all the final states and applying a more sophisticated theoretical description of the coupled-channel effect and line shapes, and so on.

### Discovery of the iso-triplet charmoniumlike *Z*_*c*_(3900) and *Z*_*c*_(4020) states

Searching for charged charmoniumlike states is one of the most promising ways of establishing the existence of the exotic hadrons, since such a state must contain at least four quarks and, thus, could not be a conventional meson. These searches have concentrated on decay final states that contain one charged pion and a charmonium state, such as *J*/ψ, ψ(2*S*) and *h*_*c*_, since they are narrow and their experimental identification is relatively unambiguous.

The first reported charged charmoniumlike state, *Z*_*c*_(4430)^−^, was found in the π^−^ψ(2*S*) invariant mass distribution in *B* → *K*π^−^ψ(2*S*) decays by the Belle experiment in 2008 [60,61]. It was confirmed by the LHCb experiment seven years later [62]. The *Z*_*c*_(3900)^−^ state was observed in the π^−^*J*/ψ invariant mass distribution in the study of *e*^+^*e*^−^ → π^+^π^−^*J*/ψ at BESIII [21] and Belle [49] experiments, and the *Z*_*c*_(4020)^−^ state was observed in the π^−^*h*_*c*_ system in *e*^+^*e*^−^ → π^+^π^−^*h*_*c*_ [63] only at BESIII.

#### Observation of the *Z*_*c*_(3900) state

**Figure 4.**
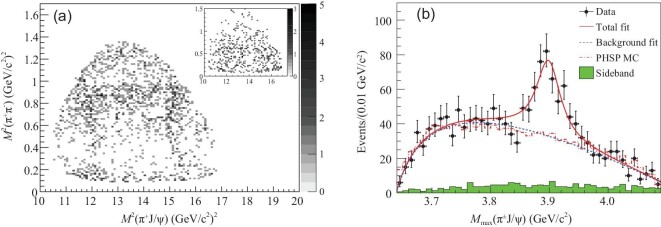
Dalitz plot for selected *e*^+^*e*^−^ → π^+^π^−^*J*/ψ events in (a) the *J*/ψ signal region (the inset shows background events from the *J*/ψ mass sidebands) and (b) the *Z*_*c*_(3900) signal in *M*_max_(π*J*/ψ) [21]. Points with error bars are data, the curves are best fits, the dashed histograms are the phase space distributions and the shaded histograms are the non-π^+^π^−^*J*/ψ background estimated from the normalized *J*/ψ sidebands.

The BESIII experiment studied the *e*^+^*e*^−^ → π^+^π^−^*J*/ψ process using a 525 pb^−1^ data sample at a c.m. energy of $4.26\,\,\rm GeV$ [21]. About 1500 signal events were observed and the cross section was measured to be (62.9 ± 1.9 ± 3.7) pb, which agrees with the previous existing results from the Belle [47] and BaBar [48] experiments. The intermediate states in this three-body system were studied by examining the Dalitz plot of the selected candidate events, as shown in Fig. 4.

In addition to the known *f*_0_(500) and *f*_0_(980) structures in the π^+^π^−^ system, a structure at around 3.9 GeV/*c*^2^ was observed in the π^±^*J*/ψ invariant mass distribution with a statistical significance larger than 8σ, which is referred to as the *Z*_*c*_(3900). A fit to the π^±^*J*/ψ invariant mass spectrum (see Fig. 4) determined its mass to be (3899.0 ± 3.6 ± 4.9) MeV/*c*^2^ and its width to be $(46\pm 10\pm 20)\,\,\rm MeV$.

A measurement performed at the Belle experiment that was released subsequent to the BESIII paper reported the observation of the *Z*_*c*_(3900) state (referred to as *Z*(3900)^+^ in the Belle paper) produced via the ISR process with a mass of (3894.5 ± 6.6 ± 4.5) MeV/*c*^2^ and a width of $(63\pm 24\pm 26)\,\,\rm MeV$ with a statistical significance larger than 5.2σ [49]. These observations were later confirmed by an analysis of CLEO-c data at a c.m. energy of $4.17\,\,\rm GeV$ [64], with a mass and width that agree with the BESIII and Belle measurements.

BESIII studied the spin-parity of the *Z*_*c*_(3900) with a partial wave analysis (PWA) of about 6000 *e*^+^*e*^−^ → π^+^π^−^*J*/ψ events at $\sqrt{s}=4.23$ and $4.26\,\,\rm GeV$ [65]. The fit indicated that the spin-parity *J*^*P*^ = 1^+^ assignment for the *Z*_*c*_(3900) is favored over other quantum numbers (0^−^, 1^−^, 2^−^ and 2^+^) by more than 7σ.

The *Z*_*c*_(3900) mass determined from its π*J*/ψ invariant mass distribution is slightly above the $D\bar{D}^*$ mass threshold. The open-charm decay $Z_c(3900)^\pm \rightarrow (D\bar{D}^*)^\pm$ was observed with much larger rate than that to π*J*/ψ [66,67], and the pole mass and width were determined with high precision to be (3882.2 ± 1.1 ± 1.5) MeV/*c*^2^ and $(26.5\pm 1.7\pm 2.1)\,\,\rm MeV$, respectively.

**Figure 5.**
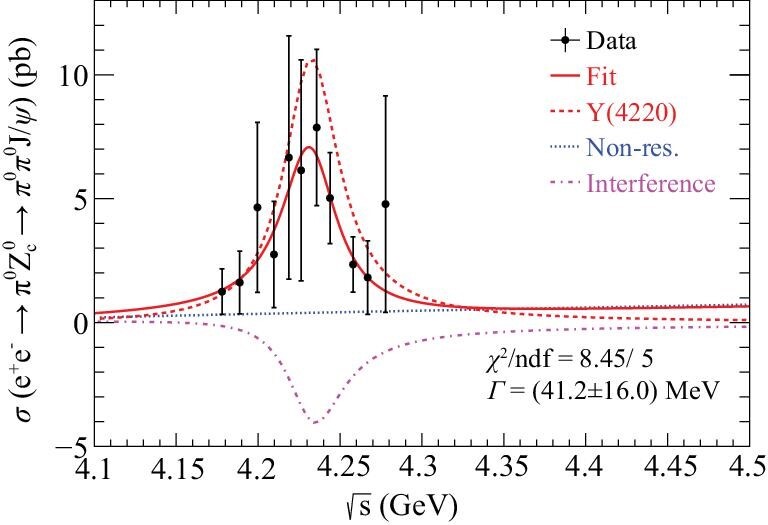
The cross sections of *e*^+^*e*^−^ → π^0^*Z*_*c*_(3900)^0^ → π^0^π^0^*J*/ψ [70]. Points with error bars are data, the red solid curve is the total fit result, the red dashed (blue dotted) curve is the resonant (non-resonant) component and the magenta dash–dot line represents the interference of the two components.

In both the QCD tetraquark and the molecular pictures, the *Z*_*c*_(3900)^±^ states are the *I*_3_ = ±1 members of an isospin triplet. BESIII confirmed this by observing their neutral, isospin *I*_3_ = 0 partners: the *Z*_*c*_(3900)^0^, in both the π^0^*J*/ψ [68] and $(D\bar{D}^*)^0$ [69] decay modes. These observations establish *Z*_*c*_(3900) as an isovector state with even *G* parity. From a PWA to the *e*^+^*e*^−^ → π^0^π^0^*J*/ψ data in the vicinity of the *Y*(4260) resonance, it is found that the cross-section line shape of *e*^+^*e*^−^ → π^0^*Z*_*c*_(3900)^0^ → π^0^π^0^*J*/ψ is in agreement with that of *Y*(4220) (see Fig. 5) [70].

BESIII also searched for the *Z*_*c*_(3900) isospin violating decay mode η*J*/ψ [71] as well as to the light hadron final states ωπ [72], $K\bar{K}\pi$ and $K\bar{K}\eta$ [73]. These modes were not observed and the upper limits of the decay rates are one order of magnitude or even smaller than that for *Z*_*c*_(3900) → π*J*/ψ, as naively expected.

#### Observation of the *Z*_*c*_(4020) state

**Figure 6.**
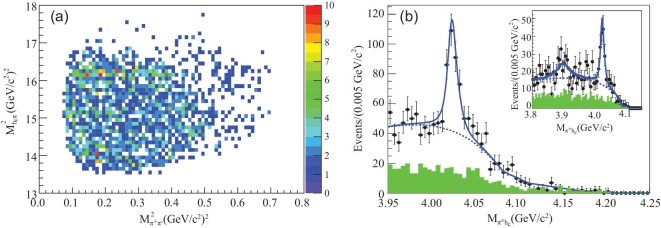
Dalitz plot ($M^2_{\pi ^+h_c}$ versus $M^2_{\pi ^+\pi ^-}$) for (a) selected *e*^+^*e*^−^ → π^+^π^−^*h*_*c*_ events and (b) the *Z*_*c*_(4020) signal observed in the π*h*_*c*_ invariant mass spectrum [63]. Points with error bars are data, the solid curves are best fits and the shaded histograms are the non-π^+^π^−^*h*_*c*_ background estimated from the normalized *h*_*c*_ sidebands.

The process *e*^+^*e*^−^ → π^+^π^−^*h*_*c*_ was observed at c.m. energies of $3.90\!-\!4.42\,\,\rm GeV$ [63] with cross section that is similar to that for *e*^+^*e*^−^ → π^+^π^−^*J*/ψ [52]. Intermediate states of this three-body system were studied by examining the Dalitz plot of the selected π^+^π^−^*h*_*c*_ candidate events, similar to what was done for the *e*^+^*e*^−^ → π^+^π^−^*J*/ψ process [21]. Although there are no clear structures in the π^+^π^−^ system, there is distinct evidence for an exotic charmoniumlike structure in the π^±^*h*_*c*_ system, as clearly evident in the Dalitz plot shown in Fig. 6. This figure also shows projections of the *M*(π^±^*h*_*c*_) (two entries per event) distribution for the signal events as well as the background events estimated from normalized *h*_*c*_ mass sidebands. There is a significant peak at around 4.02 GeV/*c*^2^ (the *Z*_*c*_(4020)), and there are also some events at around 3.9 GeV/*c*^2^ that could be due to the *Z*_*c*_(3900). The mass and width of the *Z*_*c*_(4020) were measured to be (4022.9 ± 0.8 ± 2.7) MeV/*c*^2^ and $(7.9\pm 2.7\pm 2.6)\,\,\rm MeV$, respectively. The statistical significance of the *Z*_*c*_(4020) signal is greater than 8.9σ.

In an analysis of the *e*^+^*e*^−^ → π^0^π^0^*h*_*c*_ process, the *Z*_*c*_(4020)^0^, the neutral isospin partner of the *Z*_*c*_(4020)^±^ was observed in the π^0^*h*_*c*_ system. This indicates that the *Z*_*c*_(4020) is an isovector state [74]. The open-charm decay of the *Z*_*c*_(4020) was observed in $e^+e^-\rightarrow (D^*\bar{D}^*) \pi$, with a rate that is much larger than that for its decay into π*h*_*c*_ [75,76]. Partial wave analysis of *e*^+^*e*^−^ → π^+^π^−^ψ(2*S*) is needed to confirm or deny the decay *Z*_*c*_(4020) → πψ(2*S*) [77].

#### Nature of the *Z*_*c*_ states

Although many measurements have been performed on the *Z*_*c*_(3900) and *Z*_*c*_(4020) states, the experimental information is still not very precise. From the experience of the *Z*_*c*_(4430) state, we know that the resonance parameters determined from a simple one-dimensional fit to the invariant mass distribution [60] may differ from those based on a full amplitude analysis with the interference effects between different amplitudes considered properly [61]. The same thing may happen with the *Z*_*c*_(3900) and *Z*_*c*_(4020) states. Amplitude analyses that are applied to the relevant final states that extract the resonant parameters as well as the couplings to different modes are essential to obtain more refined information for understanding the nature of these states. In addition, a PWA can also provide measurements of Argand plots of the *Z*_*c*_ amplitudes, which can be used to discriminate between different models for the *Z*_*c*_ states.

The production of *Z*_*c*_ states at a variety of c.m. energies can reveal whether these states are from resonance decays or continuum production. So far, only the *Z*_*c*_(3900) state has been observed both in *e*^+^*e*^−^ annihilation [21] and in *B*-hadron decays [78,79]. Searches for these states in different production modes is of great importance.

These states seem to indicate that a new class of hadrons has been observed. Since there are at least four quarks within each of these *Z*_*c*_ states, they have been alternatively interpreted as compact tetraquark states, molecular states of two charmed mesons ($D\bar{D}^*+D^*\bar{D}$, $D^*\bar{D}^*$, etc.), hadro-quarkonium states or other multiquark configurations; in some phenomenological studies they have been attributed to purely kinematical effects [5,15–17]. Since many of these models require assumptions that are hard to prove, it is essential that non-perturbative studies such as lattice QCD (LQCD) provide a way to understand their underlying nature; if the *Z*_*c*_ structures are not purely kinematical effects, they should appear on the lattice since they are strong interaction phenomena.

The currently available LQCD calculations that are relevant to the *Z*_*c*_(3900) state have a number of uncertainties, as has been recently reviewed in [80]. These include the lattice spacing, the volume, the physical π mass and the channels that are considered in the calculation.

An early lattice study performed by Prelovsek *et al.* [81] investigated the energy levels of two-meson systems, including π*J*/ψ, πψ(2*S*), ρη_*c*_, $D\bar{D}^*$, $D^*\bar{D}^*$, etc., as well as tetraquark operators. However, no convincing signals for extra new energy levels apart from the almost free scattering states of the two mesons were identified. Taking $D\bar{D}^*$ as the main relevant channel, the CLQCD collaboration performed a calculation that was based on the single-channel Lüscher finite-size formalism and found a slightly repulsive interaction between the two charmed mesons [82,83]. The results therefore do not support the possibility of a shallow bound state for the two mesons for the pion mass values of 300, 420 and 485 MeV/*c*^2^. A preliminary study using staggered quarks found no *J*^*PC*^ = 1^+−^ state distinct from the noninteracting scattering states either, but the authors also pointed out that future calculations with a larger interpolating operator basis may be able to resolve this state [84].

The HALQCD collaboration studied the problem using an approach where an effective potential is extracted from the lattice data and then used to solve the Schrödinger-like equations [85,86]. A fully coupled-channel potential for π*J*/ψ, ρη_*c*_ and $D\bar{D}^*$ interactions is obtained, and a strong off-diagonal transition between π*J*/ψ and $D\bar{D}^*$ indicates that the *Z*_*c*_(3900) state can be explained as a threshold cusp within their current configuration (*m*_π_ = 400−700 MeV/*c*^2^). In order to establish a definite conclusion on the structure of the *Z*_*c*_(3900) state in the real world, full QCD simulations near the physical point are being carried out [85,86].

Recently, in order to clarify the mismatch between these two approaches, CLQCD performed a two-channel lattice study using the two-channel Ross-Shaw effective range expansion [87]. They considered the π*J*/ψ and $D\bar{D}^*$ channels that are most strongly coupled to *Z*_*c*_(3900) and found that the parameters of the Ross-Shaw matrix do not seem to support the HALQCD scenario. The parameters turn out to be large and the Ross-Shaw *M* matrix is far from singular, which is required for a resonance close to the threshold. However, since only two channels are studied, it is still not a direct comparison with the HALQCD approach, in which three channels were studied. In [80], the same three channels that the HALQCD collaboration analyzed were considered, namely π*J*/ψ, ρη_*c*_ and $D\bar{D}^*$. However, the final results will not come very soon.

Whatever the nature of the *Z*_*c*_ states turns out to be, they will teach us a lot about the hadronic structures. Unless all these structures are purely kinematical effects (in which case it would have to be an as yet unknown kinematic effect), they will suggest a new category of hadrons beyond the conventional meson and baryon picture. The observation of similar states in the bottom sector [88] and recent discoveries of structures with two pairs of charm-anticharm quarks [89] and with a minimal four-quark configuration $cs\bar{u}\bar{d}$ [90] confirm this expectation. Additional searches for other conceivable states should be performed and the theoretical consequences of these new types of hadrons should be investigated.

### Comprehensive study of *X*(3872) in the *e*^+^*e*^−^ collision

The *X*(3872) was first observed in *B*^±^ → *K*^±^π^+^π^−^*J*/ψ decays in 2003 by Belle [41]. It was confirmed subsequently by several other experiments [91–94]. Prior to 2014, the *X*(3872) was only observed in *B* meson decays and hadron collisions. Since the quantum numbers of *X*(3872) are *J*^*PC*^ = 1^++^, it can be produced via radiative decays of excited vector charmonium or charmoniumlike states such as the ψ and the *Y*.

**Figure 7.**
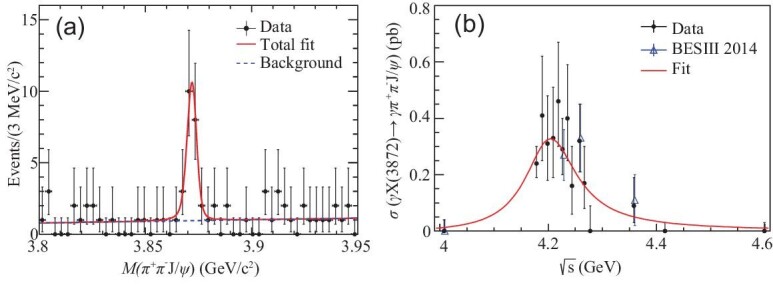
(a) Fit to the *M*(π^+^π^−^*J*/ψ) distribution [95] and (b) fit to $\sigma ^B[e^+e^-\rightarrow \gamma X(3872)]\times {\cal B}[X(3872)\rightarrow \pi ^+\pi ^- J/\psi ]$ [96]. Dots/triangles with error bars are data and the curves are best fits.

The *X*(3872) was observed at BESIII in the process *e*^+^*e*^−^ → γ*X*(3872) → γπ^+^π^−^*J*/ψ, *J*/ψ → ℓ^+^ℓ^−^ [95] (see Fig. 7(a)) and this first measurement was subsequently improved with more data [96]. The c.m. energy dependence of the product of the cross section σ[*e*^+^*e*^−^ → γ*X*(3872)] and the branching fraction ${\cal B}[X(3872)\rightarrow \pi ^+\pi ^-J/\psi ]$ is shown in Fig. 7(b), where the red curve shows the results of a fit to a BW resonance line shape with a mass of $(4200.6^{+7.9}_{-13.3}\pm 3.0)\,\,{\rm MeV}/c^2$ and a width of $(115^{+38}_{-26}\pm 12)\,\,{\rm MeV}$. These resonance parameters are consistent with those of the ψ(4160) charmonium state [3] or the *Y*(4220) (see the section entitled ‘New insights into the *Y* states’) within errors.

Using all the data samples available at c.m. energies between 4.0 and $4.6\,\,\rm GeV$, BESIII is able to observe for the first time significant signals of *X*(3872) → ω*J*/ψ [96] and *X*(3872) → π^0^χ_*c*1_ [97], and search for other possible decays.

BESIII confirmed earlier observations of a large $X(3872)\rightarrow D^{*0}\bar{D}^0+ {\rm c.c.}$ branching fraction and found evidence for *X*(3872) → γ*J*/ψ with a significance of 3.5σ [98]. No evidence was found for the decays *X*(3872) → γψ(2*S*). The upper limit on the ratio ${\cal B}(X(3872)\rightarrow \gamma \psi (2S))/ {\cal B}(X(3872)\rightarrow \gamma J/\psi ) < 0.59$ was obtained at the 90% C.L. [98], which is inconsistent with LHCb [99] and BaBar measurements [100] but consistent with a Belle upper limit [101]. No significant *X*(3872) → π^0^χ_*c*0, 2_ signals were observed.

The hadronic transitions of the *X*(3872) to low-mass charmonum states via a single pion or a rho meson violate isospin, and the large decay rates of *X*(3872) → π^0^χ_*c*1_ and *X*(3872) to ρ*J*/ψ → π^+^π^−^*J*/ψ relative to the isospin-conserved mode *X*(3872) → ω*J*/ψ indicate that *X*(3872) is very unlikely to be a pure charmonium state, such as the χ_*c*1_(2*P*). The order of magnitude larger decay rate to $D^{*0}\bar{D}^0+ {\rm c.c.}$ than to the charmonium final state favors the $D^*\bar{D}+ {\rm c.c.}$ molecule interpretation of the *X*(3872), as does the relatively smaller production rate of *X*(3872) → γψ(2*S*) compared with *X*(3872) → γ*J*/ψ, or at least that there is a large fraction of molecular component in its wave function in addition to a charmonium component.

BESIII measured the ratios of branching fractions for *X*(3872) → γ*J*/ψ, γψ(2*S*), ω*J*/ψ, π^0^χ_*c*1_, $D^{*0}\bar{D}^0+ {\rm c.c.}$, $\pi ^0D\bar{D}$ and $\gamma D\bar{D}$ to that for *X*(3872) → π^+^π^−^*J*/ψ. By combining these with the measurements of the *X*(3872) properties from the *B* factories, Li and Yuan [102] obtained the absolute branching fractions of the *X*(3872) decays into six modes by globally fitting the measurements provided by the Belle, BaBar, BESIII and LHCb experiments (see Table 2). The branching fraction for *X*(3872) → π^+^π^−^*J*/ψ is determined to be $(4.1^{+1.9}_{-1.1})\%$, which is in good agreement with earlier estimates in [103] and [104]. By combining the branching fractions of all of the observed modes, the fraction of the unknown decays of the *X*(3872) is found to be $(31.9_{-31.5}^{+18.1})\%$, which is an important challenge for future experimental studies of *X*(3872) decays.

**Table 2.** The fitting results of the absolute branching fractions of the *X*(3872) decays [102]. The branching fraction of the *X*(3872) decays into unknown modes is calculated from the fit results.

Parameter index	Decay mode	Branching fraction (%)
1	*X*(3872) → π^+^π^−^*J*/ψ	$4.1^{+1.9}_{-1.1}$
2	$X(3872)\rightarrow D^{*0}\bar{D}^0+ {\rm c.c.}$	$52.4^{+25.3}_{-14.3}$
3	*X*(3872) → γ*J*/ψ	$1.1^{+0.6}_{-0.3}$
4	*X*(3872) → γψ(2*S*)	$2.4^{+1.3}_{-0.8}$
5	*X*(3872) → π^0^χ_*c*1_	$3.6^{+2.2}_{-1.6}$
6	*X*(3872) → ω*J*/ψ	$4.4^{+2.3}_{-1.3}$
	*X*(3872) → unknown	$31.9_{-31.5}^{+18.1}$

With a very large sample of *X*(3872) → π^+^π^−^*J*/ψ events, the LHCb experiment reported an improved measurement of its mass and a first measurement of its width [105]. Limited by its capability of D^*0^ reconstruction and mass resolution, it is still not possible for LHCb to measure the line shape of the resonance.

### Commonality among the *X*(3872), *Y*(4260) and *Z*_*c*_(3900) states

With data taken with a c.m. energy at and near the *Y*(4260) resonance peak, BESIII discovered a clear signal for *X*(3872) production in association with a γ ray [95], as shown in Fig. 7, and a clear signal for *Z*_*c*_(3900) production in association with a π meson [70], as shown in Fig. 5. The c.m. energy dependence of the *e*^+^*e*^−^ → γ*X*(3872) cross section is suggestive of a *Y*(4260) → γ*X*(3872) decay process, and that of the *e*^+^*e*^−^ → π^0^*Z*_*c*_(3900)^0^ cross section is suggestive of a *Y*(4260) → π*Z*_*c*_(3900) decay process; these indicate that there might be some common features to the internal structures of the *Z*_*c*_(3900), *Y*(4260) and *X*(3872) states.

Many of the models developed to interpret the nature of one of these three states do not consider the possibility of a connection between them. With data supplied by the BESIII experiments, some of these models may be ruled out and others may need to be revisited in light of these new observations.

## SUMMARY AND PERSPECTIVES

With the capability of adjusting the *e*^+^*e*^−^ c.m. energy to the peaks of resonances, combined with the clean experimental environments due to near-threshold operation, BESIII is uniquely able to perform a broad range of critical measurements of charmonium physics, and the production and decays of many of the nonstandard $\rm XYZ$ states, as discussed above in the context of the studies of the *X*(3872), *Y*(4220), *Z*_*c*_(3900) and *Z*_*c*_(4020). Table 3 shows the operating times associated with the discoveries of the $\rm XYZ$ states at BESIII and other experiments, including the previous generation *B* factories, BaBar and Belle, and the new generation super-*B* factories, LHCb and Belle II. BESIII’s special advantages for studying the $\rm XYZ$ states are evident.

**Table 3.** The numbers of observed events of discovery modes of the $\rm XYZ$ states at BESIII and other experiments. Here the states are detected with *X*(3872) → π^+^π^−^*J*/ψ, *Y*(4260) → π^+^π^−^*J*/ψ, *Z*_*c*_(3900)^±^ → π^±^*J*/ψ, *Z*_*c*_(4020)^±^ → π^±^*h*_*c*_ and *Y*(4660) → π^+^π^−^ψ(2*S*). The numbers for the Belle II experiments are a simple scale according to those of the Belle experiment. Here ‘–’ indicates not available. BESIII can detect other decay modes of these states while other experiments can barely do so.

Experiment	Data-taking time	*X*(3872)	*Y*(4260)	*Z*_*c*_(3900)	*Z*_*c*_(4020)	*Y*(4660)
BESIII	3 months	20	6000	1300	180	250
BaBar	1999–2008	90	270	80	–	45
Belle	1999–2010	170	550	160	–	90
LHCb	2011–12 (*B* decays)	4000	–	–	–	–
	2011–18 (*pp* collision)	16 000	–	–	–	–
Belle II	2019–30	8000	28 000	8000	–	5000

We emphasize here that BESIII measured all the known decay modes of the *X*(3872) and discovered its new decay modes even though the numbers of produced *X*(3872) events are much smaller than those of other experiments. This is because the very clean experimental environment of *e*^+^*e*^−^ collisions in the τ-charm threshold energy region uniquely facilitates the isolation of signals for *X*(3872) decays into final states with one or more photons with high efficiency. This is especially true for final states that contain an *h*_*c*_ charmonium state like the BESIII discovery of the *Z*_*c*_(4020) state and measurements of *Y*(4220) and *Y*(4390) → π^+^π^−^*h*_*c*_ decays. Neither the BaBar and Belle *B* factories nor the LHCb experiment has ever seen an *h*_*c*_ signal.

BESIII has produced a considerable amount of information about the $\rm XYZ$ and the conventional charmonium states. In addition, there are data that are still being analyzed and more data that will be accumulated at other c.m. energies [56,106]. Analyses with these additional data samples will provide an improved understanding of the $\rm XYZ$ states, especially the *X*(3872), *Y*(4260), *Z*_*c*_(3900), and *Z*_*c*_(4020) states. The maximum c.m. energy accessible at BEPCII was upgraded from 4.6 to 5.0 GeV in 2019, and a 5.6 fb^−1^ of data was accumulated in the 2019-20 and 2020–21 running periods, with more data planned for the future. This enables a full coverage of the *Y*(4660) [44] resonance and a search for possible higher mass vector mesons and states with other quantum numbers, as well as improved measurements of their properties.

At the same time, other experiments will also supply information on these states. At the LHCb, in addition to the 3 fb^−1^ of data at 7 and 8 TeV that have been used for most of their published analyses, there is a 6 fb^−1^ of data sample at 13 TeV that is being used for improved analyses of many of the topics discussed above such as the *X*(3872) decay properties and searches for the *Y* and *Z*_*c*_(3900) states in *B* decays.

Belle II [107] has collected about 200 fb^−1^ of data by mid-2021, and will accumulate 50 ab^−1^ data at the ϒ(4*S*) peak by the end of 2030. These data samples can be used to study the $\rm XYZ$ and charmonium states in many different ways [14], among which ISR can produce events in the same energy range covered by BESIII. A 50 ab^−1^ Belle II data sample will correspond to 2.0–2.8 fb^−1^ of data for every 10 MeV from 4–5 GeV. Similar statistics will be available for modes like *e*^+^*e*^−^ → π^+^π^−^*J*/ψ at Belle II and BESIII (after considering the fact that Belle II has lower efficiency). Belle II has the advantage that data at different energies will be accumulated at the same time, making the analysis much simpler than at BESIII.

There are two super τ-charm factories proposed, the STC in China [108] and the SCT in Russia [109]. Both machines would run at c.m. energies of up to 7 GeV with a peak luminosity of 10^35^ cm^−2^ s^−1^, which is a factor of 100 improvement over the BEPCII. This would enable systematic studies of the $\rm XYZ$ and charmonium states with unprecedented precision.
